# Modelling the Regulation of Thermal Adaptation in *Candida albicans*, a Major Fungal Pathogen of Humans

**DOI:** 10.1371/journal.pone.0032467

**Published:** 2012-03-20

**Authors:** Michelle D. Leach, Katarzyna M. Tyc, Alistair J. P. Brown, Edda Klipp

**Affiliations:** 1 School of Medical Sciences, University of Aberdeen, Aberdeen, United Kingdom; 2 Theoretische Biophysik, Humboldt-Universität, Berlin, Germany; King's College London Dental Institute, United Kingdom

## Abstract

Eukaryotic cells have evolved mechanisms to sense and adapt to dynamic environmental changes. Adaptation to thermal insults, in particular, is essential for their survival. The major fungal pathogen of humans, *Candida albicans*, is obligately associated with warm-blooded animals and hence occupies thermally buffered niches. Yet during its evolution in the host it has retained a *bona fide* heat shock response whilst other stress responses have diverged significantly. Furthermore the heat shock response is essential for the virulence of *C. albicans*. With a view to understanding the relevance of this response to infection we have explored the dynamic regulation of thermal adaptation using an integrative systems biology approach. Our mathematical model of thermal regulation, which has been validated experimentally in *C. albicans*, describes the dynamic autoregulation of the heat shock transcription factor Hsf1 and the essential chaperone protein Hsp90. We have used this model to show that the thermal adaptation system displays perfect adaptation, that it retains a transient molecular memory, and that Hsf1 is activated during thermal transitions that mimic fever. In addition to providing explanations for the evolutionary conservation of the heat shock response in this pathogen and the relevant of this response to infection, our model provides a platform for the analysis of thermal adaptation in other eukaryotic cells.

## Introduction

Stress adaptation is essential for the survival of all organisms. In particular, the heat shock response is a fundamentally important process that has been highly conserved from yeasts to humans. In response to a sudden and acute temperature up-shift, cells rapidly induce the expression of genes that encode molecular chaperones, proteases and other classes of protein [1]. These proteins function in the synthesis, folding, maturation, trafficking and degradation of proteins, and are essential for protection against, and recovery from the cellular damage associated with the presence of the aberrantly folded proteins generated by the heat shock [2], [3], [4].

In eukaryotic cells the expression of heat shock protein (*HSP*) genes is controlled by the heat shock transcription factor [5], [6], which is evolutionarily conserved from *Saccharomyces cerevisiae* (Hsf1) to humans (HSF1/2). *S. cerevisiae* Hsf1 is an essential protein that binds to heat shock elements (HSEs) in the promoter regions of target genes, which include *HSP* genes. Hsf1 activation leads to the up-regulation of these target genes in response to heat shock [7], [8] thereby promoting cellular adaptation to the thermal insult.

The major fungal pathogen of humans, *Candida albicans*, has retained a heat shock response [9], even though this yeast is obligately associated with warm-blooded animals [10], [11]. Like *S. cerevisiae, HSP* gene activation in *C. albicans* is mediated by an essential, evolutionarily conserved heat shock transcription factor, Hsf1 [12]. It is thought that, via this heat shock regulon, *C. albicans* cells tune the levels of essential chaperones to their ambient growth temperature [9]. *C. albicans* appears to be well adapted to its human host. It exists as a relatively harmless commensal organism within the microbial flora of the oral and gastrointestinal tracts in many individuals [13]. However, it often causes mucosal infections in otherwise healthy individuals (*thrush*), and can instigate life-threatening systemic infections in immunocompromised patients [10], [11]. Indeed, approximately 40% of haematogenously disseminated *Candida* infections are fatal in some patient groups [14], [15], [16].

Historically, the heat shock response in *C. albicans* has been of interest for a number of reasons. First, temperature up-shifts promote morphological transitions from the yeast to hyphal growth forms [17], [18], and this cellular morphogenesis is a major virulence trait in *C. albicans* [19], [20], [21]. Second, mutations that block Hsf1 activation in *C. albicans* prevent thermal adaptation and significantly reduce the virulence of this major pathogen [12]. Third, antifungal drug resistance is abrogated both by Hsp90 inhibitors and by elevated temperatures equivalent to those in febrile patients [22]. Fourth, *C. albicans* heat shock proteins are immunogenic, thereby directly affecting host-pathogen interactions during infection [23], [24]. Finally, autoantibodies against Hsp90 are immunoprotective against *C. albicans* infections [25], [26], [27]. Taken together, the heat shock response of fungal pathogens is of fundamental importance because it is essential for virulence [12], and because heat shock proteins represent targets for novel therapeutic strategies [28].

The exact mechanisms by which thermal adaptation is regulated in eukaryotic cells have been extensively studied, but are still not yet fully understood. When human cells are exposed to heat or a chemical stress, protein unfolding increases, and non-native proteins begin to accumulate [29], [30], [31]. These non-native proteins are believed to compete with HSF1 for binding to Hsp90, resulting in an increase in unbound HSF1 molecules which rapidly trimerize [32], [33]. In yeast, when cells are exposed to an acute thermal stress, proteins unfold, the heat shock transcription factor becomes activated by phosphorylation [9], and this induces the expression of heat shock genes [34]. However, key questions remain unanswered in fungi. For example, do heat shock proteins play a role in regulating the heat shock response, for instance possibly by down-regulating Hsf1 following stress adaptation? Almost three decades ago, Lindquist [35] and Didomenico *et al.* [36] postulated that feedback components exist to down-regulate the heat shock response. Initially, Hsp70 was proposed to be a key repressor of Hsf1 activation [37], [38], [39], [40], [41], but later evidence indicated that Hsp70 is in fact a prerequisite for Hsp90-dependent functions [42]. Indeed, a role for Hsp90 in Hsf1 repression was suggested following the observation that pharmacological inhibition of Hsp90 correlates with HSF1 activation in mammalian cells [33], [43]. Zou and colleagues demonstrated that HSF1 can be cross-linked to Hsp90 in unstressed HeLa cells, suggesting that HSF1 might interact with Hsp90 [33]. Additionally, the trimeric form of human HSF1 has been shown to associate with an Hsp90-immunophilin-p23 complex, and this is thought to repress HSF1 transcriptional activity [44]. Furthermore, HSP90 modulates HSF1 regulation in *Xenopus* oocytes [45]. In yeast, mutations that interfere with Hsp90 function have been shown to derepress the expression of Hsf1-dependent reporter genes in *S. cerevisiae* [46]. These data infer the existence of an autoregulatory loop in yeast, whereby Hsf1 activates *HSP90* expression, and then Hsp90 down-regulates Hsf1 activity. How could this autoregulatory loop control the dynamics of heat shock adaptation over time?

The functionality of biological systems depends upon both negative and positive feedback loops, such that system inputs reinforce or oppose the system output, respectively. Systems biology approaches are being increasingly utilised as a tool to examine the functionality, behaviour and dynamic properties of complex biological systems. However, despite the fundamental importance of heat shock regulation, the application of mathematical modelling to this adaptive response has been very limited. A few studies have examined the robustness of bacterial heat shock systems, which involve the transcriptional control of heat shock functions by the sigma factor σ^32^ [47], [48]. Also, there has been minimal modelling of heat shock systems in eukaryotic cells. Rieger and co-workers examined the regulation of *HSP70* gene transcription by HSF1 in response to heat shock in cultured mammalian cells [49]. Meanwhile Vilaprinyo and co-workers modelled the metabolic adaptation of yeast cells to heat shock [50]. However, there has been no mathematical examination of the relationship between Hsp90 and Hsf1 in any system. Furthermore, few dynamic models have been reported for any molecular systems in *C. albicans* or other fungal pathogens. Yet it is clear that mathematical modelling will provide useful complementary approaches to the experimental dissection of these organisms, and will help to accelerate our progress in elucidating how pathogens adapt to the complex and dynamic microenvironments they encounter in their human host.

Modelling biochemical networks allows the integration of experimental knowledge into a logical framework to test, support or falsify hypotheses about underlying biological mechanisms. Indeed, modelling can emphasise holistic aspects of systems which can often disappear in the experimental dissection of individual components of large systems. Moreover, when a model has been established, it can be used to further test hypotheses, or simulate behaviours that would be difficult to test in the laboratory. We reasoned that a combination of mathematical modelling and experimental dissection will enhance our understanding of how pathogens adapt to the temperature shifts they encounter in febrile patients, for example.

Therefore, in this study we have exploited an integrative systems biology approach to study the dynamic regulation of the heat shock response in *C. albicans*. Our model was constructed around the assumption that an autoregulatory loop involving Hsf1 and Hsp90 plays a central role in the control of thermal adaptation. The model was parameterised using experimental data that defined the dynamics of the heat shock response in this pathogen. The model was then utilised to make well-defined predictions about the behaviour of this system that were subsequently confirmed experimentally. This has allowed us to draw several important conclusions. In particular we have shown that the heat shock system displays so-called perfect adaptation [51], in that Hsf1 activation returns to basal levels following adaptation to a new ambient temperature. We also predicted and then confirmed experimentally how the system responds to sequential thermal insults, or stepwise increases in temperature. In this way our mathematical modelling has provided important insights into the behaviour of an invading fungal pathogen under physiologically relevant but experimentally intransigent conditions.

## Results

### Development of a dynamic model of heat shock adaptation in *C. albicans*

With a view to understanding the conserved and dynamic mechanisms by which organisms control thermal adaptation, we firstly constructed a predictive mathematical model of the heat shock response using a number of assumptions (stated below). This model focuses on the interaction between Hsf1 and Hsp90. This is because while other chaperones (such as Hsp70) were initially thought to repress HSF1 [37], [38], [39], [40], [41], more recent experimental evidence has indicated that Hsp90 is the major repressor of mammalian HSF1 [33], [43]. We do not exclude the possibility that other molecules may contribute to this regulation. However, for the sake of simplicity, only the major repressor (Hsp90) is included in our model. In brief, the model describes the temporal changes of components involved in the mechanism with ordinary differential equations (ODE). Every process that alters the concentration of a compound enters the right hand side of the ODE with either a positive (producing) or negative (degrading reaction) sign. These processes are nonlinear and coupled, and thus their evolution is not predictable from intuition, but requires simulation. Having constructed the model we parameterised it using experimental data generated for tractable heat shocks *in vitro*. We then exploited this model to examine thermal adaptation during sequential and stepwise thermal insults as well as during less tractable temperature fluctuations that occur *in vivo*.

Several assumptions were made in the initial construction of this model. First, we assumed that Hsp90 interacts with and negatively regulates Hsf1 under steady state conditions, in the absence of thermal fluctuation [46]. Second, we reasoned that in response to heat shock, proteins become unfolded, that Hsp90 becomes sequestered in complexes with these unfolded proteins, and that this leads to the release of Hsf1 from Hsp90-Hsf1 complexes. Third, we assumed that free Hsf1 becomes phosphorylated and activated by its protein kinase, leading to the induction of heat shock protein genes including *HSP90* [9], [52]. Fourth, we predicted that this protein kinase is down-regulated by an unknown inhibitor. Fifth, on the basis that Hsp90 negatively regulates Hsf1 (assumption 1), we predicted that the subsequent increase in Hsp90 levels would then lead to the down-regulation of Hsf1.

**Figure 1:**
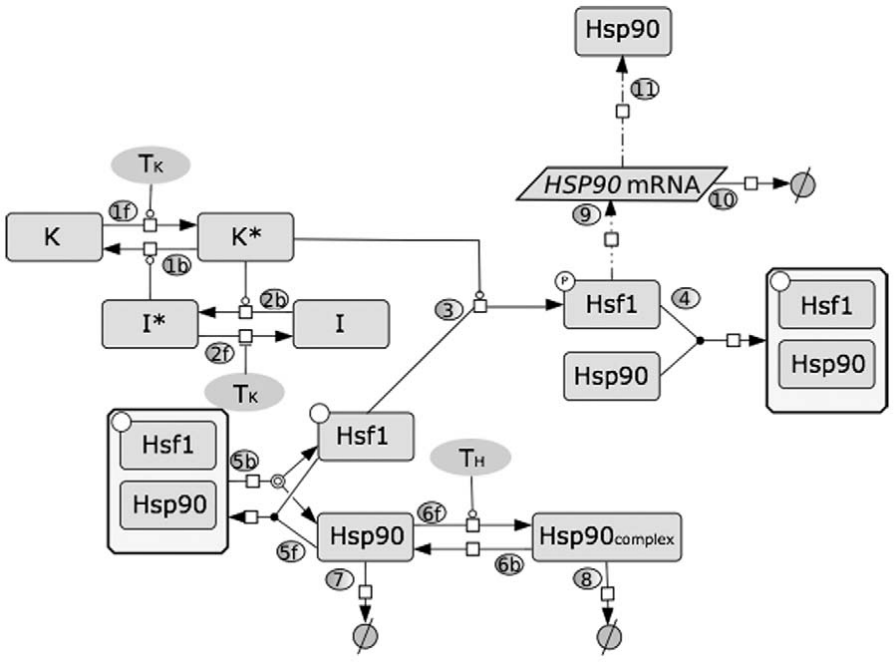
Model depicting heat shock adaptation in *C. albicans*. In this minimal molecular model of heat shock response the arrows represent mass flow or chemical reaction. The numbers in circles specify the respective step in the model. The lines ended with circles indicate positive regulation and lines with blind ends indicate negative regulation. Dashed arrows represent transcription (9) or translation (11) of *HSP90*. See text for detailed description of the molecular mechanism: Hsp90_complex_, Hsp90 complexes with unfolded proteins; K and K*, inactive and active forms of an activating protein kinase; I and I*, inactive and active forms of an inactivating protein phosphatase.

Our goal was to keep the mathematical model as simple as possible, reducing the complexity of the system to include the following key components: the inactive (unphosphorylated) and active (phosphorylated) forms of Hsf1; the interaction of Hsf1 with Hsp90; free Hsp90; the Hsp90 complex with unfolded proteins; and *HSP90* mRNA production (Figure 1). Therefore, we considered three main forms of Hsp90: the free form (Hsp90), the complex with unfolded proteins (Hsp90Complex) and the complex with Hsf1 (Hsf1Hsp90). We made this assumption on the basis that: (a) molecular chaperones participate in the (re)folding of many proteins in eukaryotic cells [2], [3], [4]; (b) in mammalian cells, unfolded proteins accumulate during heat shock [29], [30], [31]; and (c) these unfolded proteins are thought to compete with HSF1 for binding to Hsp90, leading to the release of free HSF1 [32], [33]. Therefore, we proposed that Hsf1 is present in an equilibrium with Hsp90, constantly associating with and dissociating from Hsp90 (5f; 5b). At elevated temperatures the protein kinase(s) that phosphorylates Hsf1 (K) becomes activated (K*) (1f), and this leads to the subsequent activation of an inhibitor I* (2b) which inactivates K* (1b). The identities of the Hsf1 kinase(s) (K/K*) and Hsf1 phosphatase(s) (I/I*) are currently unknown. The active K* binds free Hsf1, forming the complex Hsf1K* (3), mediating Hsf1 phosphorylation to form Hsf1P (3). Activated Hsf1 (Hsf1P) induces the transcription of *HSP90* mRNA via heat shock elements within promoter regions (9), and subsequently induces the synthesis of new Hsp90 (11). The model also accounts for the degradation of *HSP90* mRNA (10). The transcriptional activity of Hsf1P can be repressed through the binding of Hsp90 and the formation of the complex Hsf1Hsp90 (4). Thus Hsf1 is assumed to be negatively regulated by Hsp90 in the model. During heat shock, Hsp90 (together with other chaperones in the heat shock protein families) binds unfolded and/or damaged proteins, preventing their aggregation and helping them to refold (6f) [53]. This is considered a reversible process (6b). In addition, both the Hsp90Complex (8) and Hsp90 can be degraded (7). The degradation of Hsp90 protein and *HSP90* mRNA are not explicitly regulated by heat shock in the model. However, the increased formation of Hsp90Complex due to a temperature up-shift indirectly promotes Hsp90 degradation by affecting the equilibrium between free and Hsf1-bound Hsp90. The initial conditions, the ODEs that define this model, and the parameter values are presented in Tables 1, 2, 3, and 4. Details about how the initial conditions and parameter values were determined are provided in the section on *Modelling reaction kinetics and parameter estimation* in the Materials and Methods.

**Table 1:** Notations used throughout.

Definition	Comment	Initial Value
K	Inactive protein kinase	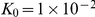
K*	Active protein kinase	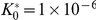
I	Inactive inhibitor	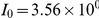
I*	Active inhibitor	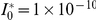
Hsp90	Heat Shock Protein Hsp90	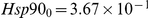
Hsp90Complex	Hsp90 bound to other unfolded proteins	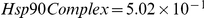
Hsf1Hsp90	Hsp90 coupled with Hsf1, mainly available before the stress	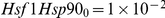
Hsf1	Heat shock transcription factor Hsf1	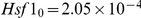
Hsf1P	Phosphorylated Hsf1	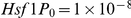
*HSP90*mRNA	*HSP90* mRNA	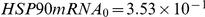

**Table 2:** Ordinary Differential Equations used in the model.

ODEs
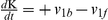
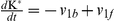
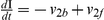
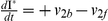
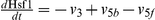
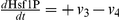
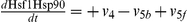
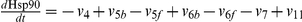
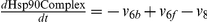
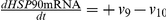

**Table 3 pone-0032467-t003:** Kinetic rate laws used in the model.

Kinetic rate laws
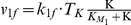
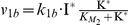
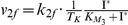
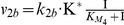
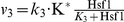
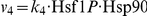
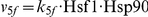
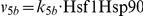
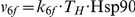
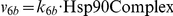
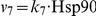
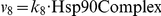
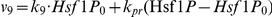
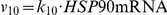
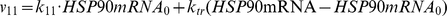

**Table 4 pone-0032467-t004:** Parameter values and assumptions.

Parameter Value	Comment
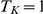 , 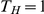	30°C
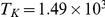 , 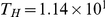	37°C
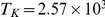 , 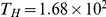	42°C
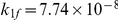 , 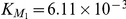	Rate constant of protein kinase activation and Michaelis Menten constant
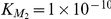	Michaelis Menten constant for protein kinase deactivation
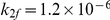 , 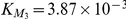	Rate constant of inhibitor activation and Michaelis Menten constant
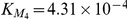	Michaelis Menten constant for inhibitor deactivation
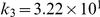 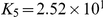	Hsf1 phosphorylation rate constantAnd Michaelis Menten constant
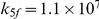	Rate constant of Hsf1 and Hsp90 association
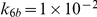	Rate constant dissociation of Hsp90 from Hsp90Complex
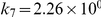	Rate constant of Hsp90 degradation
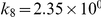	Rate constant of Hsp90Complex degradation
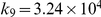	Basal *HSP90*mRNA production
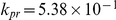	Rate constant of *HSP90*mRNA production
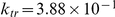	Rate constant of Hsp90 protein production
**Steady state assumption:**	
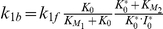	Rate constant of K* to K transition
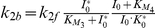	Rate constant of I to I* transition
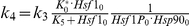	Hsf1 dephosphorylation rate constant
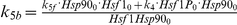	Rate constant of Hsf1 dissociation from the complex Hsf1Hsp90
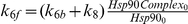	Rate constant of Hsp90 binding to unfolded proteins
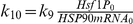	*HSP90*mRNA degradation rate constant
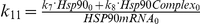	Basal production of Hsp90 protein

### Dynamics of heat shock adaptation in *C. albicans*

**Figure 2:**
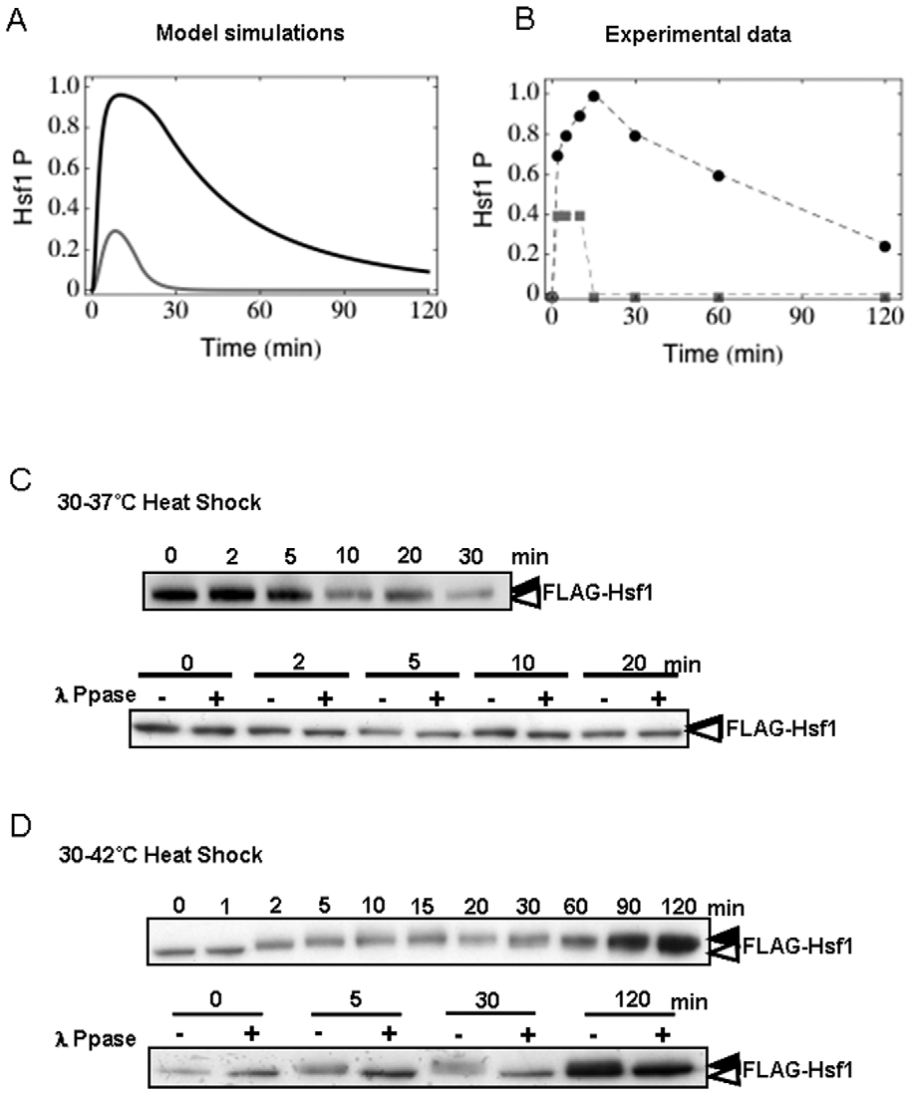
Dynamic changes in Hsf1 phosphorylation during the heat shock response. Comparison of model simulations and experimental evidence depicting Hsf1activation during heat shock. (A) Model simulations of the dynamic changes in Hsf1 phosphorylation levels following heat shock: grey line 30°C–37°C, black line: 30°C–42°C. (B) Graphical illustration of experimentally determined Hsf1 phosphorylation levels following heat shock from experiments such as those shown in (C) and (D): grey line 30°C–37°C, black line: 30°C–42°C. (C) The upper panel shows Hsf1 phosphorylation levels during a 30°C–37°C heat shock. The lower panel shows lambda phosphatase controls (λ Ppase) that confirm that Hsf1 is phosphorylated at the 2, 5 and 10 minute time points in the upper panel. (D) The upper panel shows Hsf1 phosphorylation levels during a 30°C–42°C heat shock. The lower panel shows lambda phosphatase controls (λ Ppase) that confirm Hsf1 phosphorylation for the 5, 30 and 120 minute time points in the upper panel. White and black arrows depict the unphosphorylated and phosphorylated forms respectively. Data reflect the outcomes for at least three independent replicate experiments.

**Figure 3:**
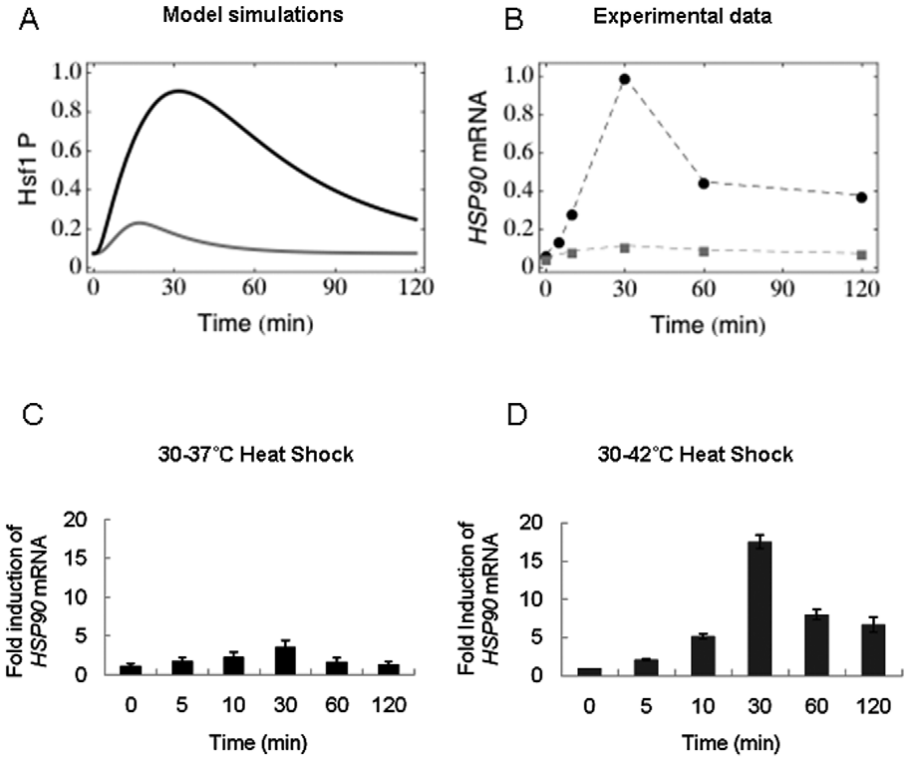
Dynamic changes in *HSP90* mRNA during the heat shock response. Comparison of model simulations and experimental evidence representing *HSP90* mRNA levels during thermal upshifts. (A) Model simulations of the dynamic changes in *HSP90* mRNA levels following heat shock: grey line 30°C–37°C, black line: 30°C–42°C. (B) Graphical illustration of experimentally determined *HSP90* mRNA levels following heat shock generated in experiments such as those shown in (C) and (D): grey line 30°C–37°C, black line: 30°C–42°C. (C) qRT-PCR quantification of *HSP90* mRNA levels relative to the internal *ACT1* mRNA control were analysed during a 30°C–37°C heat shock. (D) qRT-PCR quantification of *HSP90* mRNA levels relative to the internal *ACT1* mRNA control were analysed during a 30°C–42°C heat shock.

Having constructed the model, it was parameterised to fit the experimentally determined dynamics of thermal adaptation in *C. albicans*. (For more information about the parameter fitting see the Materials and Methods section.) These included the kinetics of Hsf1 phosphorylation, (a proxy for Hsf1 activation [9]) (Figure 2) and the temporal induction of *HSP90* mRNA levels (Figure 3) during 30°C–37°C and 30°C–42°C heat shocks.

Replicate time series measurements of Hsf1 phosphorylation were completed for both 30°C–37°C and 30°C–42°C heat shocks (Figures 2 and 3). Protein extracts were prepared, subjected to western blotting, and Hsf1 phosphorylation levels quantified (Figure 2B). Lambda phosphatase controls were run routinely to confirm band-shifts representing Hsf1 phosphorylation (Figures 2C and 2D). Low levels of Hsf1 phosphorylation were reproducibly detected during a 30°C–37°C heat shock (Figure 2C). These subtle band-shifts were resolvable by lambda phosphatase at 2, 5 and 10 minutes post heat shock, but no band-shifts were observed after 10 minutes indicating that by 20 minutes Hsf1 phosphorylation levels had returned to basal levels equivalent to the no heat shock controls (Figure 2C). Hsf1 phosphorylation levels were assayed up to 120 minutes post heat shock, but no detectable phosphorylation was observed after 20 minutes (not shown). In contrast, cells that received a 30°C–42°C heat shock routinely displayed strong levels of Hsf1 phosphorylation within two minutes, the activation continuing to increase up to 20 minutes post heat shock before starting to decline again (Figure 2D). Hsf1 phosphorylation levels had returned to low levels by the 120 minute time point. Once again, the band-shifts corresponding to Hsf1 phosphorylation were confirmed by the lambda phosphatase controls (Figure 2D). These observations were reproducible in multiple independent experiments.

*HSP90* mRNA levels were also measured experimentally. During a 30°C–37°C heat shock, *HSP90* mRNA levels increased approximately three-fold relative to the internal *ACT1* mRNA control (Figure 3C), whereas *HSP90* mRNA levels increased approximately sixteen-fold in response to a 30°C–42°C heat shock (Figure 3D). This was experimentally consistent with the stronger Hsf1 phosphorylation observed during a 30°C–42°C heat shock. Furthermore the peaks of *HSP90* mRNA followed after the peaks of Hsf1 activation (Figure 2). Similar observations were made in three independent experiments.

Following parameterisation the model simulated the experimentally determined dynamics of Hsf1 phosphorylation and *HSP90* mRNA induction with reasonable accuracy. The simulations predicted the rapid and transient phosphorylation of Hsf1 during 30°C–37°C and 30°C–42°C heat shocks (Figure 2A – grey and black lines, respectively). Furthermore, the model correctly predicted that during a 30°C–37°C heat shock, the amplitude of Hsf1 phosphorylation is lower and of a shorter duration than during a 30°C–42°C heat shock (Figure 2A). In addition, the model correctly predicted that *HSP90* mRNA levels are induced about four-fold more strongly during a 30°C–42°C heat shock compared with a 30°C–37°C heat shock (Figure 3A).

Our model does not include Hsf1 production. This is because we considered the dynamics of thermal adaptation over a 120 minute timescale, which corresponds to less than two generations of growth under our experimental conditions. We have shown previously that Hsf1 levels change after protracted growth of *C. albicans* at different temperatures [46]. However, in this study we did not observe significant changes in Hsf1 levels over the 120 minute timescale examined. Before excluding Hsf1 production from the model we tested the theoretical impact of Hsf1 production upon the dynamics of the system. To achieve this we conceptually doubled the amount of Hsf1 present in the cell. Interestingly, this did not change the dynamics of Hsf1 phosphorylation during a 30°C–42°C heat shock, the concentration of phosphorylated Hsf1 always tending to zero after 120 minutes.

### Sensitivity analyses

We performed sensitivity analyses to investigate the sensitivity of the system during the adaptation to thermal challenges. A classical approach to sensitivity analysis (Metabolic control analysis: MCA) can be used to assess infinitesimally small changes in individual reactions (parameter values or the initial concentrations of regulators and enzymes) influence the steady state concentrations in the model. MCA was initially founded to investigate metabolic networks but is now also used to examine the sensitivity of signalling pathways or gene regulatory networks [54]. In order to address specifically the influence of parameter choice upon the dynamics of our system, we used time-varying response coefficients that allowed us to test responses to individual parameter perturbations along the entire trajectory rather than its influence on a steady state only.

By studying time-varying response coefficients we examined whether there are single reactions or parameters that greatly influence the dynamics of the thermal adaptation system. We used the mathematical formalism to describe firstly the non-scaled response coefficients [55].


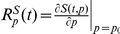
, with 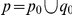 and *p_0_* being a set of parameters employed in the model and *q_0_* being a set of initial conditions.

Our point of reference was the nominal trajectory of Hsf1 phosphorylation over time simulated with the model following exposure to a 30°C–42°C thermal shock.

We perturbed every parameter from the set *p* and studied the impact upon the Hsf1 phosphorylation trajectory over a 120 minute period. A positive value for the response coefficient 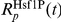 indicated a temporal or sustained increase in the relevant substrate concentration _(Hsf1P)_ as the value of the parameter *p* was increased, whereas a negative value for 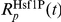 translated into a decrease in the Hsf1 phosphorylation level. To compare the response coefficients they were scaled (and hence dimensionless) according to the formula:


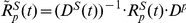
, where 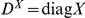 is a diagonal matrix [55].

**Figure 4:**
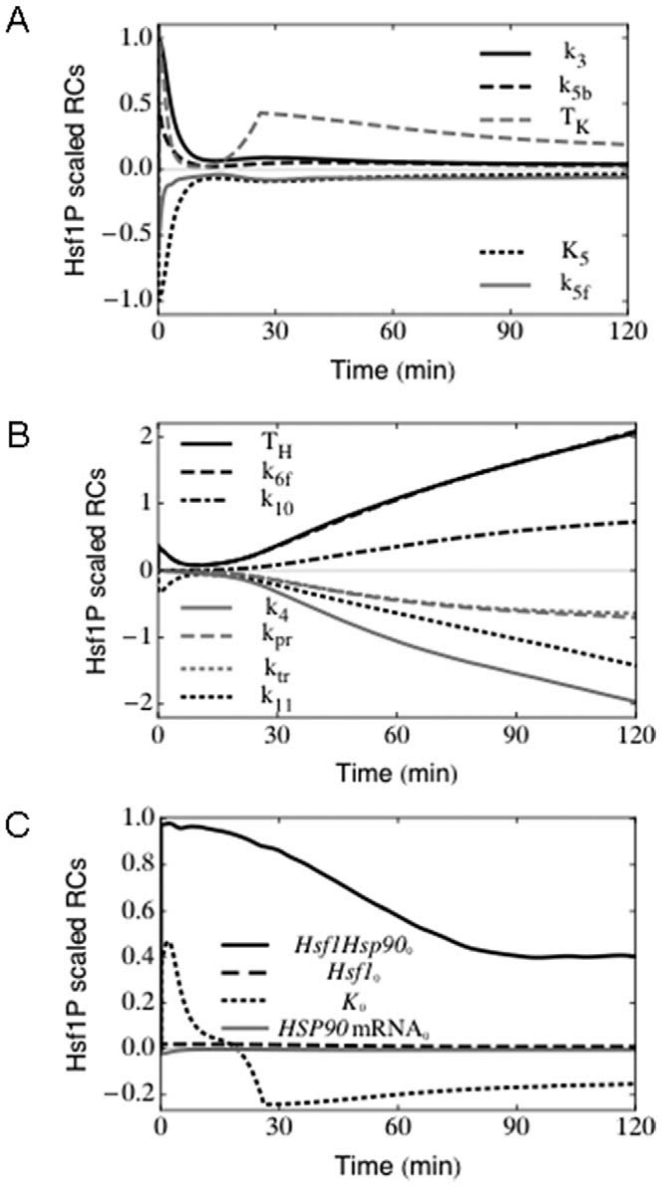
Sensitivity analyses. Selected time-varying response coefficients for phosphorylated Hsf1 for a 37°C–42°C heat shock are presented. The parameters not presented in this figure had negligible influence upon the Hsf1 phosphorylation dynamics over the 120 minute timescale examined. (A) Early response coefficients are shown. These influence the Hsf1 phosphorylation dynamics during the first ∼10 minutes, but do not significantly affect thermal adaptation in the longer term. (B) Late response coefficients. These mainly influence Hsf1 phosphorylation dynamics after ∼15 minutes leading to differences in the concentration of phosphorylated Hsf1 at the 120 minute time point. For instance, the parameter T_H_ has a positive response coefficient at 120 minutes, which means that an increment in its numerical value will result in increased accumulation of phosphorylated Hsf1 at 120 minutes. (C) Response coefficients calculated for the initial concentrations. An increased initial concentration of the Hsf1-Hsp90 complex leads to higher Hsf1 phosphorylation levels throughout the period of thermal adaptation examined.

Selected non-zero response coefficients are presented in Figure 4. As expected, perturbations in the phosphorylation reaction (reaction 3) increased the initial level of phosphorylated Hsf1 (Hsf1P), but the response after 120 minutes essentially remained unchanged (Figure 4A). In contrast, an increase in the rate of association of Hsp90 with Hsf1P, which leads to its dephosphorylation (parameter 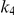), resulted in a negative 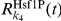 value for t = 120 minutes (Figure 4B). In other words, increasing the value of 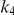 leads to lower levels of Hsf1 phosphorylation at 120 minutes. (This would be the case during the whole adaptation process as the value of 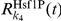 was negative for all 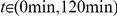. It is also worth noting that 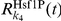 is a late response coefficient in that it impacts upon the dynamics of the system about 15 minutes after the application of the heat shock (Figure 4B).

Interestingly, the initial concentration of free Hsf1 did not influence the final result of the adaptation (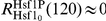). One might have assumed that having more free Hsf1 in the system would result in higher Hsf1 phosphorylation levels at 120 minutes, but this was not the case, which was counter intuitive (Figure 4C). The same held for the initial concentration of free Hsp90, which gave a 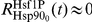, for all 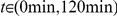. One could expect to have lower Hsf1 phosphorylation levels throughout the trajectory, which again was not the case.

In summary in our thermal adaptation model, the parameters that had the most impact upon Hsf1 phosphorylation dynamics were those involved in the reactions describing the complex formation between Hsp90 and Hsf1P (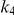), the interaction between Hsp90 with other malfunctioning proteins (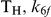), and Hsp90 translation itself (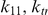). All the other parameters either influenced Hsf1 phosphorylation dynamics to a low extent (for instance for *HSP90* mRNA processing, 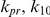), or to minimal extents within the considered timescale (the remaining parameters).

### Impact of sequential heat shocks

Having demonstrated that this model simulates the molecular responses of *C. albicans* during thermal adaptation with reasonable accuracy, we then exploited the model to predict molecular responses under other conditions. It is well known in a variety of systems including mammalian cells [56], [57], *Drosophila* cells [58], [59], *S. cerevisiae* [60], [61], *Dictyostelium* sp. [62], plant cells [63] and bacteria [64], [65] that an initial treatment with a mild heat shock leads to an increased resistance to subsequent exposure to otherwise lethal temperatures. This phenomenon is termed acquired thermotolerance. A related phenomenon has also been demonstrated in *C. albicans*, whereby a prior heat shock protects cells against a high oxidative stress [66], [67]. This phenomenon infers that the thermal adaptation system retains a molecular “memory” of the first heat shock that leads to the protection of cells against the second heat shock. From a molecular perspective we predicted that this might be reflected in reduced Hsf1 phosphorylation during a second heat shock. We tested this prediction using our model to simulate the effects of exposing cells to sequential heat shocks.

**Figure 5:**
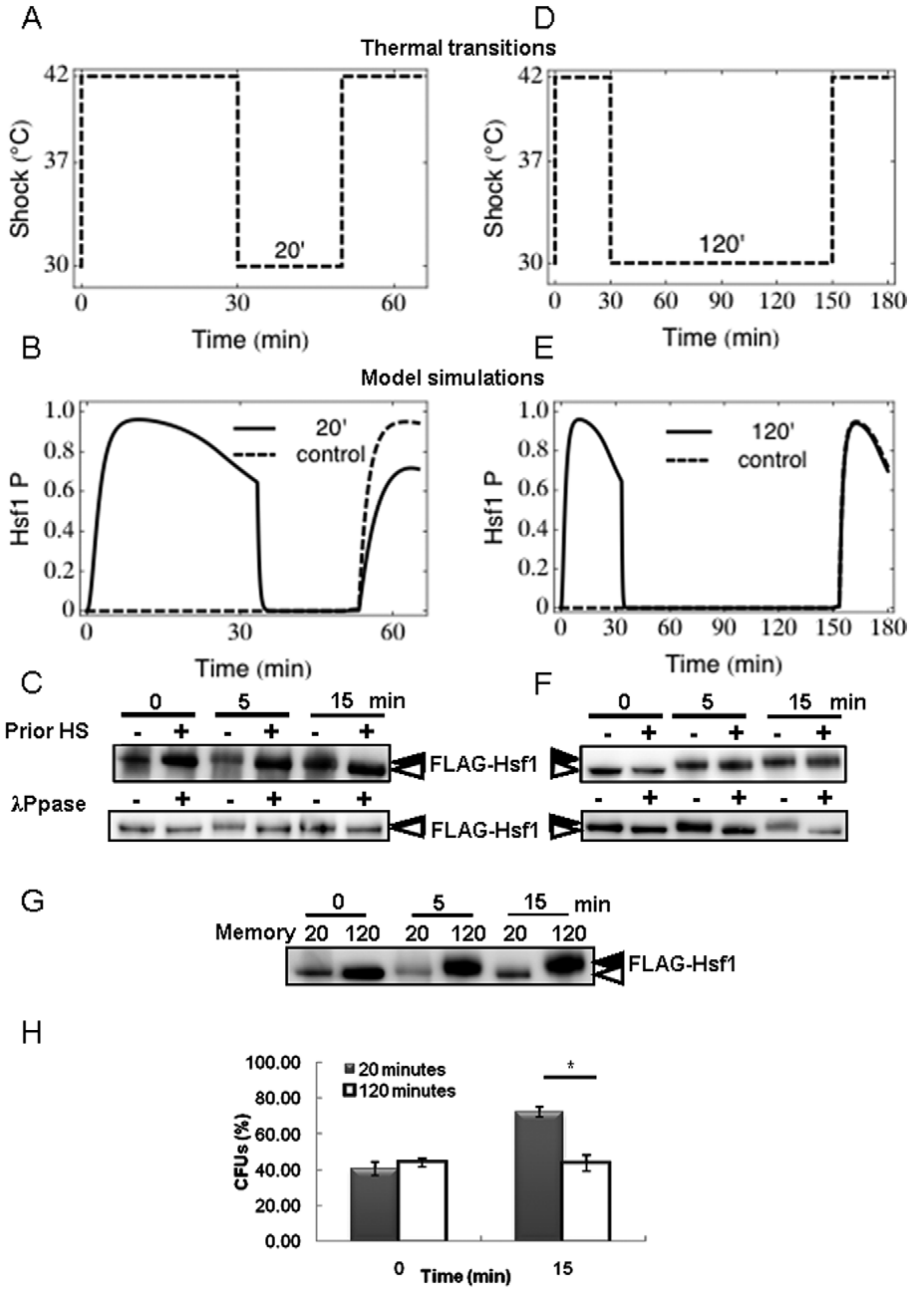
Impact of sequential heat shocks. The model was used to simulate sequential 30 minute heat shocks of 30°C–42°C separated either by 20 or 120 minutes. Outcomes were then tested experimentally by determining Hsf1 phosphorylation levels. (A) Representations of the thermal transitions with the 20 minute recovery period. (B) Model simulations of Hsf1 phosphorylation for a 20 minute recovery period between heat shocks: solid black line simulates Hsf1 phosphorylation in cells that have seen a prior heat shock, dashed black line simulates Hsf1 phosphorylation in control cells that have had no prior heat shock. (C) These predictions were tested experimentally by moving exponentially growing cells from 30°C to 42°C for 30 minutes. Cells were then placed back at 30°C for 20 minutes before they received a second heat shock at 42°C. Control cells only received the second heat shock with no prior heat shock. Proteins were extracted and subjected to western blotting to measure Hsf1 phosphorylation (upper panel). The lower panel shows lambda phosphatase controls (λ Ppase) that confirm the Hsf1 phosphorylation states shown in the upper panel. (D) Representations of the thermal transition with a 120 minute recovery period between heat shocks. (E) Model simulation of Hsf1 phosphorylation for a 120 minute recovery period between heat shocks: solid black line, predicted Hsf1 phosphorylation in cells that have seen a prior heat shock; dashed black line, predicted Hsf1 phosphorylation in control cells that have had no prior heat shock. (F) These predictions were tested experimentally by moving exponentially growing cells from 30°C to 42°C for 30 minutes. Cells were then placed back at 30°C for 120 minutes before they received a second heat shock at 42°C. Control cells only received the second heat shock with no prior heat shock. Protein extracts were subjected to western blotting to measure Hsf1 phosphorylation (upper panel). The lower panel includes lambda phosphatase controls (λ Ppase) that confirm the phosphorylation status of samples from the upper panel. (G) Further controls involving direct comparison of 20 and 120 minute memory samples to confirm the differential Hsf1 phosphorylation in these samples, and hence the loss of molecular memory after 120 minutes. (H) The loss of molecular memory is reflected in reduced cellular resistance to the second heat shock. Cell viabilities (CFUs) were measured 0 and 15 minutes after the imposition of the second 30°C–42°C heat shock: grey bars, cells that received a 20 minute interval between heat shocks; grey bars, cells that received a 120 minute interval between heat shocks (* p<0.05, students t-test). Data reflect the outcomes for at least two independent replicate experiments.

Using the model, cells were exposed to a conceptual heat shock of 30°C–42°C for 30 minutes. They were then placed back at 30°C for a specific time interval (20 or 120 minutes) before subjecting them to a second 30°C–42°C heat shock (Figure 5A). Two main predictions arose from these simulations. Firstly, the model predicted that, with an interval of 20 minutes between heat shocks, Hsf1 does become rephosphorylated during the second heat shock, albeit not to the same extent as control cells that have not received a prior heat shock (Figure 5B). This unexpected prediction was then tested experimentally. As predicted, significant Hsf1 band shifts were routinely observed in cells following a second 30°C–42°C heat shock. Once again, lambda phosphatase controls were included confirming that these band shifts represented Hsf1 phosphorylation (Figure 5C). Also as predicted, the levels of Hsf1 phosphorylation were reproducibly reduced compared with control cells that did not receive the prior heat shock.

A second important prediction was that this memory would be lost when the period between 30°C–42°C heat shocks was extended to 120 minutes (Figure 5D). Under these conditions, the model predicted that the levels of Hsf1 phosphorylation during the second heat shock would be equivalent to Hsf1 phosphorylation levels in control cells that do not receive a prior heat shock (Figure 5E). Once again this prediction was confirmed experimentally (Figure 5F). We consistently observed an equivalent Hsf1 band shift between samples whether or not the cells had received a prior heat shock. As before, we confirmed that this band shift was due to Hsf1 phosphorylation by treating control samples with lambda phosphatase (Figure 5F). Furthermore to highlight the loss of molecular memory following the extended interval between heat shocks, the samples for the 20 minute and 120 minute intervals were compared alongside each other. There was a striking difference in their Hsf1 phosphorylation levels (Figure 5G) further confirming that the molecular memory observed after a 20 minute interval between heat shocks was lost after 120 minutes.

The transient nature of the molecular memory was tested further in the laboratory by comparing the viabilities of *C. albicans* cells that received different regimes of sequential heat shocks (Figure 5H). Cells that received a prior 30°C–42°C heat shock 30 minutes before the second heat shock displayed a significant increase in survival in comparison with control cells that did not receive a prior heat shock. In contrast, and as predicted by the model, this increase in survival was no longer observed in cells that received the prior heat shock 120 minutes before the second heat shock (Figure 5H). A number of factors could contribute to the loss of viability, including a reduction in the levels of heat shock proteins following thermal adaptation, and/or a decrease in the protectant trehalose [68]. However, the model correctly predicted that the molecular memory, and hence acquired thermotolerance, is transient. This memory protects cells against sequential heat shocks that occur within a short period, but is lost if subsequent heat shocks occur after this period.

### Impact of stepwise heat shocks

As discussed above, the acute heat shocks that tend to be studied *in vitro* do not accurately reflect the thermal fluctuations that cells generally encounter in the wild. Therefore, we used the model to examine the effects of temperature changes that are more closely related to those in the wild. The generation of fevers is a primary response of patients to systemic candidiasis. These fevers expose *C. albicans* cells to temperature fluctuations from approximately 37°C up to about 42°C. Therefore we used the model to examine such changes. Our first step was to predict the effects of stepwise temperature increases that are experimentally tractable.

**Figure 6:**
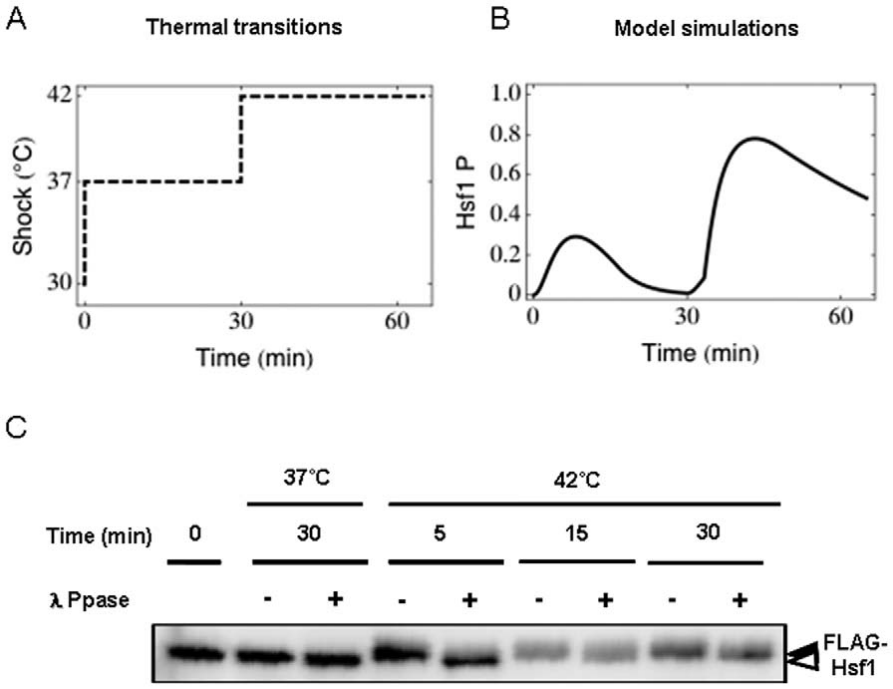
Impact of stepwise heat shocks. The model was used to simulate stepwise thermal transitions from 30°C (the starting condition) to 37°C, and then from 37°C to 42°C. Outcomes were then tested experimentally by determining Hsf1 phosphorylation levels. (A) Representations of the thermal transitions. (B) Model simulation of Hsf1 phosphorylation during stepwise 30°C–37°C and 37°C–42°C heat shocks. (C) These predictions were tested experimentally by moving exponentially growing cells from 30°C to 37°C for 30 minutes, and then transferring them from 37°C to 42°C for 30 minutes. Proteins were extracted at various time intervals and Hsf1 phosphorylation measured by western blotting. Hsf1 was shown to be transiently induced during the 37°C–42°C transitions. Lambda phosphatase (λ Ppase) controls were included to confirm that Hsf1 phosphorylation had returned to basal levels 30 minutes after a 30°C–37°C heat shock (see Figure 2), but that Hsf1 is rapidly phosphorylated following the next 37°C–42°C heat shock. The data reflect the outcomes for at least two independent replicate experiments.

The model was used to predict the impact of moving cells firstly to 37°C from 30°C (the starting condition in the model) and then allowing them to adapt to this new ambient temperature for 30 minutes before shifting them from 37°C to 42°C (Figure 6A). The model predicted that once cells have adapted to an ambient temperature of 37°C, they would still display rapid Hsf1 phosphorylation following a relatively minor thermal upshift from 37°C to 42°C (Figure 6B). This prediction was then tested experimentally (Figure 6C). Firstly, these experiments reconfirmed that Hsf1 phosphorylation is transiently induced during a 30°C–37°C heat shock, returning to basal levels within 20 minutes (compare Figure 6C: 37°C 30 minutes with Figure 2C). Then when these *C. albicans* cells were subjected to the second thermal step from 37°C–42°C, Hsf1 phosphorylation was shown to be transiently induced for a second time, as predicted by the model (Figure 6C: 42°C 5 minutes). These reproducible observations were validated with lambda phosphatase controls, which resolved Hsf1 band shifts caused by phosphorylation. Therefore, once again, novel predictions generated by the model were confirmed experimentally.

**Figure 7:**
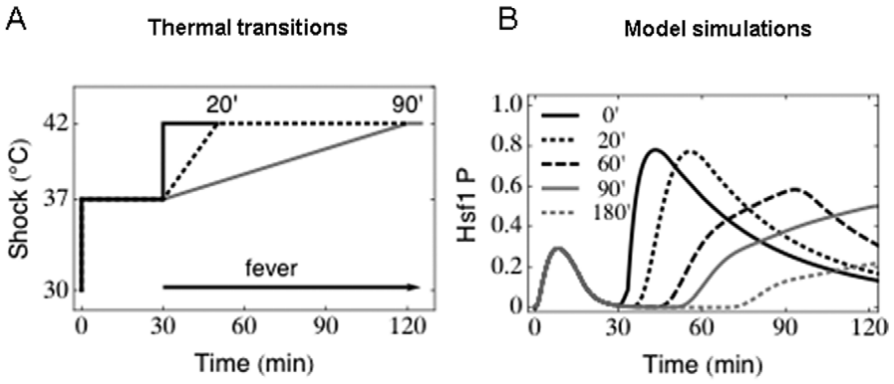
The impact of slow thermal upshifts. The model was used to simulate slow thermal transitions from 37°C–42°C over 20, 60, 90 or 180 minute periods after a 37°C heat shock for 30 minutes. (A) Representations of a subset of the thermal transitions examined, some of which might mimic fevers in patients. (B) Model simulations of Hsf1 phosphorylation levels during slow temperature transitions from 37°C–42°C: 0 minute transition, solid black line; 20 minute transition, dotted black line; 60 minute transition, dashed black line; 90 minute transition, solid grey line; 180 minute transition, dotted grey line.

Having confirmed the validity of our model, our next goal was to exploit this model to examine thermal adaptation scenarios that are more clinically relevant but not as easy to test experimentally. In particular we were interested in the molecular responses of this system to slow thermal transitions from 37°C–42°C that more closely reflect the onset of fevers in patients. Following adaptation to the initial thermal step from the initial condition to 37°C, we simulated slow thermal transitions from 37°C to 42°C over 20, 60, 90 or 180 minute periods (Figure 7A). Interestingly, Hsf1 was predicted to become phosphorylated even during these slow temperature transitions (Figure 7B). This would suggest that Hsf1 activation is required for the types of thermal adaptation that are encountered *in vivo*. This suggestion is consistent with our experimental observations. We have shown previously that mutations that block Hsf1 activation attenuate the virulence of *C. albicans* in a mouse model of systemic candidiasis [12].

### The thermal adaptation system displays perfect adaptation

A biological system is described as displaying “perfect adaptation” when its steady-state output is independent of the steady-state input. Osmoadaptation in *S. cerevisiae* provides an excellent example of this because Hog1 signalling returns to basal levels once cells have adapted to new ambient conditions [51]. According to our computational simulations the thermal adaptation system is predicted to display perfect adaptation because Hsf1 phosphorylation declines to basal levels once cells have adapted to a new ambient temperature (Figures 2A and 6B). This prediction has been confirmed experimentally (Figures 2B 2C and 2D). For example, when a 30°C–37°C heat shock is applied the system recovers rapidly, with Hsf1 phosphorylation returning to basal levels within 20 minutes (Figures 2B and 2C). After a 30°C–42°C heat shock the system recovers after about 2 hours (Figures 2B and 2D). Therefore, the heat shock regulatory network adapts perfectly to changes in ambient temperature.

This observation is significant because it accounts for another experimental result that was not initially obvious. Previously we were surprised to find that while Hsf1 is phosphorylated in response to mild heat shocks, Hsf1 is not phosphorylated in *C. albicans* cells that have adapted to different growth temperatures ranging from 15°C–40°C [9]. However, the perfect adaptation displayed by the thermal adaptation system can account for this experimental result. According to the model, Hsf1 is rapidly and transiently activated when cells are adapting to a temperature upshift. However Hsf1 phosphorylation returns to basal levels once this adaptation to the new ambient temperature is complete.

## Discussion

The heat shock response is a fundamentally important adaptive response that is highly conserved across eukaryotic systems. A key function of this response is to maintain protein quality and homeostasis in the face of thermal insults through the induction of genes encoding chaperones and components of the protein degradation machinery. Our current understanding of heat shock adaptation is primarily based on a strong experimental platform that has largely focussed on dramatic and acute heat shocks [6], [69], [70], [71]. In this study we have exploited an integrative systems biology approach with a view to understanding thermal adaptation under conditions that are more relevant physiologically but less tractable experimentally. This represents one of the first applications of mathematical modelling to understand a dynamic adaptive response in a fungal pathogen.

Our mathematical model of thermal adaptation was based on a number of assumptions and upon published observations from our own and other laboratories. A previous model focused on the feedback regulatory mechanisms of heat shock proteins on HSF function in mammalian systems [49]. However, these models did not address Hsp90 specifically, although experimental inhibition of Hsp90 has been shown to activate Hsf1 in mammalian cells and yeast [33], [46]. Therefore, our model was based on the hypothesis that an autoregulatory loop involving Hsf1 and Hsp90 lies at the heart of thermal adaptation. Our experimentally verified assumption that Hsp90 is a major player in thermal adaptation does not exclude the possibility that other factors probably contribute to thermal adaptation (such as other chaperones or trehalose, for example). Furthermore, our model includes interactions between Hsf1 and its protein kinase(s) and phosphatase(s). The identity of the protein kinase(s) that phosphorylate Hsf1 in yeast is not known, and relatively little is known about the signalling cascades that trigger Hsf1 activation. The phosphatase(s) activity appears to be equally important in regulating thermal adaptation, as Hsf1 is rapidly dephosphorylated in *C. albicans* upon a cold shock (Leach and Brown, unpublished). The model accurately predicted numerous molecular behaviours of the thermal adaptation system, suggesting that our underlying hypotheses are valid (Figures 2, 3, 5 and 6). Nevertheless, in the future, the boundaries of our model could be extended to include, for example, other molecules that might contribute to the regulation of Hsf1 [42], [43], [44], [46], additional genes that are controlled by Hsf1 [8], and downstream metabolic events that contribute to thermal adaptation [50].

Interestingly, the thermal adaptation system displays perfect adaptation. Perfect adaptation, which represents a fundamental property of this system, has been reported for other eukaryotic systems and in prokaryotes [51], [72], [73], [74], [75]. This perfect adaptation accounts for a previous observation that was not intuitively obvious and remained unexplained at the time, namely that while Hsf1 is phosphorylated in response to heat shock, no Hsf1 phosphorylation is observed in cells that have had time to adapt to elevated growth temperatures [9].

For a system to display perfect adaptation it must contain a negative feedback loop [75]. In the thermal adaptation system this negative feedback loop is manifested by the Hsp90-Hsf1 autoregulatory loop, which represents a central component of the model (Figure 1). There is considerable experimental evidence for this regulatory loop in diverse systems. For example, Hsf1 is known to activate *HSP90* in yeasts [9], and Hsp90 down-regulates Hsf1 in mammalian cells and yeasts [33], [46]. The predictive accuracy of our model reinforced the validity of this autoregulatory loop.

The model was able to account for the well-known phenomenon of “induced thermotolerance”, whereby prior exposure to a heat shock protects cells against a subsequent heat shock [56], [57], [58], [59], [60], [61], [62], [63], [64], [65]. The prevailing view is that this phenomenon is mediated by the up-regulation of chaperone proteins that are retained by the cell for a certain period before being degraded. Our simulations of sequential heat shocks correctly predicted that *C. albicans* cells retain a molecular memory for a short period, and that this memory is transient, being lost within two hours. Indeed, these data fit with a decrease in *HSP90* mRNA levels, as well as Hsf1 phosphorylation 120 minutes after the imposition of a 30°C–42°C heat shock. Therefore, components of the essential machinery that would normally be present only 20 minutes after a prior heat shock appear to have been (partially) degraded after 120 minutes, meaning that if the system is subjected to a second thermal insult at this point, the cell must remount a “normal” heat shock response in order to adapt and survive. This was confirmed by assaying the viability of *C. albicans* cells that were exposed to sequential heat shocks separated by differing time intervals (Figure 4C). This infers that the chaperone Hsp90 plays a key role in the phenomenon of induced thermotolerance.

We also exploited our model to examine thermal adaptation during step-wise heat shocks, extending this to examine responses to slow temperature transitions that are more reminiscent of fevers in patients with systemic candidiasis. Interestingly the model indicates that Hsf1 is phosphorylated during slow temperature transitions and the system is still able to adapt perfectly. It is not possible to impose slow temperature transitions upon mice, and hence to test this experimentally *in vivo*. Nevertheless, these predictions were entirely consistent with our published work, which revealed that although Hsf1 is not phosphorylated during growth at elevated temperatures [9], the inability to phosphorylate Hsf1 attenuates the virulence of *C. albicans* [12]. These observations were difficult to rationalise at the time, but have been explained by our modelling of thermal adaptation (Figure 5C). Our observations can also account for the conservation of the heat shock response in a fungal pathogen that occupies thermally buffered niches.

In conclusion, the success of the model in predicting key aspects of observed molecular responses highlights the utility of systems approaches to understand biological behaviours and regulatory modules in particular. This integrative systems biology approach has provided valuable insights into the dynamic behaviours of the thermal adaptation system in *C. albicans* under conditions that are relevant to its lifestyle as a fungal pathogen that is obligately associated with warm-blooded animals. Given the highly conserved nature of the heat shock response, we suggest that our model will prove useful for the analysis of thermal adaptation in other eukaryotic systems, including mammalian cells.

## Materials and Methods

### Strains and growth conditions

*C. albicans* strains (Table 5) were grown in YPD [76]. To impose a heat shock, cells were grown to exponential phase in YPD at 30°C, and then added to pre-warmed flasks at either 37°C or 42°C together with an equal amount of pre-warmed medium at either 44°C or 54°C, thereby imposing rapid heat shocks of 30°C–37°C or 30°C–42°C, respectively.

**Table 5:** *C. albicans* strains.

Strain	Genotype	Source
THE1	*ade2::hisG/ade2::hisG, ura3::λ imm434/ura3::λ imm434, ENO1/eno1::ENO1-tetR-ScHAP4AD-3XHA-ADE2*	[86]
ML250	*ade2::hisG/ade2::hisG, ura3::*l *imm434/ura3::*l *imm434, ENO1/eno1::ENO1-tetR-ScHAP4AD-3XHA-ADE2 HSF1/HSF1, pACT1-FLAG-HSF1*	this study

### Western blotting

Total soluble protein was extracted and subjected to Western blotting using published protocols [77]. Briefly, cells were resuspended in 250 µl lysis buffer (0.1 M Tris-HCl, pH 8, 10% glycerol, 1mM DTT, pepstatin A, Protease Inhibitor Cocktail) and sheared with glass beads in a Mini-bead beater (6×30 s with 1 minute intervals on ice). Lysates were centrifuged at 13000 rpm for 10 minutes at 4°C. Proteins (15 µg) were separated by SDS-polyacrylamide gel electrophoresis (SDS-PAGE) using the XCell *SureLock*™ Mini-Cell system (Invitrogen: Paisley, UK) with NuPAGE®Novex Bis-Tris 4–12% pre-cast gels (Invitrogen) in NuPAGE® MOPS-SDS Running Buffer (Invitrogen) containing NuPAGE® Antioxidant (Invitrogen) as per the manufacturer's instructions. Proteins were transferred to Invitrolon™ PVDF Membranes (Invitrogen) in NuPAGE® Transfer Buffer containing methanol using the XCell II™ Blot Module (Invitrogen) as per the manufacturer's instructions. Following transfer, the membranes were rinsed in PBS and blocked in PBS-T+5% milk [PBS 0.1% Tween-20, 5% (w/v) milk] for 1 hour at room temperature. The membranes were then incubated for 1 hour at room temperature in PBST+5% milk containing antibody. To detect FLAG-Hsf1, a 1∶10000 dilution of anti-FLAG HRP conjugated antibody was used (Sigma A8592: Gillingham, UK). Membranes were incubated for 1 hour at room temperature. Membranes were washed in PBS-T and signals detected using an ECL Western blotting kit (Amersham: Little Chalfont, Buckinghamshire, UK) as per the manufacturer's instructions.

### mRNA analyses

*HSP90* mRNA levels were measured by qRT-PCR. *C. albicans* cells were grown in YPD at 30°C to mid-log phase (OD_600_ = 0.5), harvested and frozen rapidly in liquid N_2_. RNA was extracted with Triazol (GibcoBRL: Grand Island, NY) as described previously [78], and RNA integrity assayed on an Agilent Bioanalyser 2100 (Stockport, UK). For qRT-PCR, samples were incubated at room temperature for 15 minutes in a 20 µl reaction mix containing 2 µg RNA, 2 µl DNase I buffer (Invitrogen), 1.5 µl DNase I and 1.5 µl RNase OUT (Invitrogen). cDNA was prepared using Superscript II (Invitrogen) as per the manufacturer's protocol. Realtime PCR was performed in triplicate in optical multiwall plate 384 using the LightCycler 480 Probes Master (Roche Applied Science; Burgess Hill, UK) as previously [79], [80]. Briefly, probes were chosen for the target transcripts, *HSP90* and *ACT1*, using the ProbeFinder Software Version 2.45 (Roche, www.universalprobelibrary.com). PCR was performed in a 20 µl reaction containing 10 µl LightCycler 480 Probes Master Mix, 3 µl of 1∶5 diluted cDNA, 0.2 µl of forward and reverse primers, 0.2 µl selective probe (Roche) and 6.4 µl water, PCR grade. Negative controls were performed using water instead of cDNA. Reactions were performed in a LightCycler 480 system (Roche Applied Science) using the following parameters: preincubation at 95°C for 5 minutes, 50 cycles of amplification at 95°C for 10 seconds and 60°C for 30 seconds, and a final cooling at 40°C for 1 minute. Standard curves were prepared using four dilutions of the control, wild type. *HSP90* mRNA levels were normalised against the *ACT1* mRNA (in arbitrary units).

### Model formulation and assumptions

The mathematical model considers the relationship between Hsf1 and Hsp90. During the course of model development the following assumptions were made based on data for Hsf1 and Hsp90:

(1) Constant concentration of [Hsf1]_TOTAL_ = [Hsf1Hsp90]+[Hsf1]+[Hsf1P]
(whereby Hsf1Hsp90 represents the Hsf1-Hsp90 complex, Hsf1 represents unphosphorylated Hsf1, and Hsf1P represents phosphorylated Hsf1);
(2) We assumed the system to be in a homeostatic state before heat shock, which allowed us to set the model to the steady state for unstressed conditions;
(3) The following relation holds: *Hsf1Hsp90*_0_ *<Hsp90*_0_ *<Hsp90Complex*_0_;
*Hsp90*_0_ *≈10 Hsf1Hsp90*_0_;
*Hsp90*_0_ *≈0.6 Hsp90Complex*_0_.
(whereby Hsp90 represents free Hsp90, and Hsp90Complex represents Hsp90 that is complexed with unfolded proteins);
(4) Before stress, the amount of inactive kinase is approximately the same as the amount of Hsf1 coupled with Hsp90, *K*_0_ *≈Hsf1Hsp90*_0_.

### Analysis of the system

When modelling temperature changes, we assumed that an active protein kinase, K*, accumulates during heat shock, which leads to Hsf1 phosphorylation. The activation of K during a heat shock is characterised by the parameter T_K_. Additionally, upon temperature elevation, proteins unfold causing sequestration of Hsp90, thereby forming Hsp90 complexes with these unfolded proteins that help them to refold. It is unlikely that both events, K* accumulation and Hsp90 binding, occur with the same rate constant. This allowed us to introduce the parameter T_H_, which describes this process of Hsp90 complex formation. Hence, when simulating a heat shock, we perturbed the system through changes of the parameters T_K_ and T_H_. For different heat shocks, T_K_ and T_H_ have different numerical values, which were determined based on the response of the system (Table 2).

### Modelling reaction kinetics and parameter estimation

For modelling we used a set of ordinary differential equations (ODEs) with mass action or Michaelis Menten kinetics for the individual reactions (Tables 3–4). Initially, in our model we had 22 unknown kinetic parameters. We assumed the system to be in a steady state before a stress is imposed, and thus the number of unknown parameters was reduced to 15. Information about the total number of molecules per cell for the proteins Hsp90 and Hsf1 is not available for *C. albicans*, and therefore we estimated the initial conditions from the model together with other parameters, using the tool COPASI [81]. In this estimation process we included a range for the parameter search space for every variable in the model with the help of the yeast GFP Fusion Localization Database (http://yeastgfp.ucsf.edu) [82]. We set these ranges estimating the ratio of *Hsf1Hsp90*_0_:*Hsp90*_0_:*Hsp90Complex*_0_ to be conserved at 1∶10∶100. In addition, we assumed low numbers of molecules per cell for free *Hsf1*_0_, *Hsf1P*_0_, K*_0_, *I*_0_ and I*_0_ (with respect to *Hsf1Hsp90*_0_). We used the tool COPASI and its in built Evolutionary Programming algorithm against the experimental data to estimate these parameters [81]. The algorithm minimized the sum of squared differences between model simulation results and experimental data [83].

The initial conditions, the system of ODEs and the parameter values are given in Tables 2–5. The full model is available in systems biology markup language (SBML) file and the model annotation was done with use of semanticSBML [84]. For presenting computational results we used Mathematica7 [85].
